# Advances in small extracellular vesicles: roles in the tumor microenvironment and epithelial ovarian cancer diagnosis and treatment

**DOI:** 10.3389/fonc.2025.1526944

**Published:** 2025-02-11

**Authors:** Liang Peng, Yi Lai, Baodi Cao

**Affiliations:** ^1^ Department of Gynecology, The Second People’s Hospital of Jingdezhen, Jingdezhen, Jiangxi, China; ^2^ Department of Laboratory Medicine, Yiwu Hospital Affiliated to Hangzhou Medical College, Yiwu, Zhejiang, China

**Keywords:** small extracellular vesicles, epithelial ovarian cancer, tumor microenvironment, diagnostic biomarkers, therapeutic strategies, recent advances

## Abstract

Epithelial ovarian cancer (EOC), one of the most prevalent subtypes of ovarian cancer, has a 5-year survival rate of less than 30%, highlighting the urgent need for novel diagnostic and therapeutic strategies. The tumor microenvironment (TME), a critical regulator of tumor progression, includes various components, among which small extracellular vesicles (sEVs) serve as important molecular carriers, having gained attention as significant contributors to cancer biology. These vesicles, released by cells into the extracellular space, are pivotal in the pathogenesis of EOC. In addition, sEVs show significant promise as biomarkers and therapeutic agents for the treatment and management of this malignancy. This review explores recent advancements in the understanding of sEVs within the TME and their potential applications in the diagnosis and treatment of EOC.

## 1 Introduction

Epithelial ovarian cancers (EOCs), which account for 80-90% of all ovarian cancer cases, are the most common ovarian malignancy. Due to the complexity of its pathogenesis, deep pelvic location, and nonspecific early clinical manifestations, the 5-year survival rate remains dismally low, with minimal improvement over recent decades. Consequently, EOCs are the leading cause of death among gynecological cancers (1).

The tumor microenvironment (TME) is a complex network comprised of tumor cells, endothelial cells, stromal cells, and immune cells. The TME is key to cancer progression and tumors cannot survive without proper support from microenvironment-derived factors (2). Small extracellular vesicles (sEVs), membrane-bound structures measuring 30-100 nm in size and originating from cell endosomes, facilitate intercellular communication and play a critical role in regulating the TME. These vesicles significantly influence tumor behavior by modulating cell-cell interactions (3).

Recent studies have identified abnormal sEVs secretion in EOCs, which may alter TME dynamics (4). In addition, sEVs isolated from the plasma and tissue of EOC patients may serve as potential biomarkers for early detection and therapeutic targeting (5). This review delves into the role of sEVs in the pathogenesis and advancement of ovarian cancer by shaping the epithelial ovarian cancer microenvironment, providing new perspectives for clinical diagnosis and treatment.

## 2 Overview of sEVs

**Figure 1:**
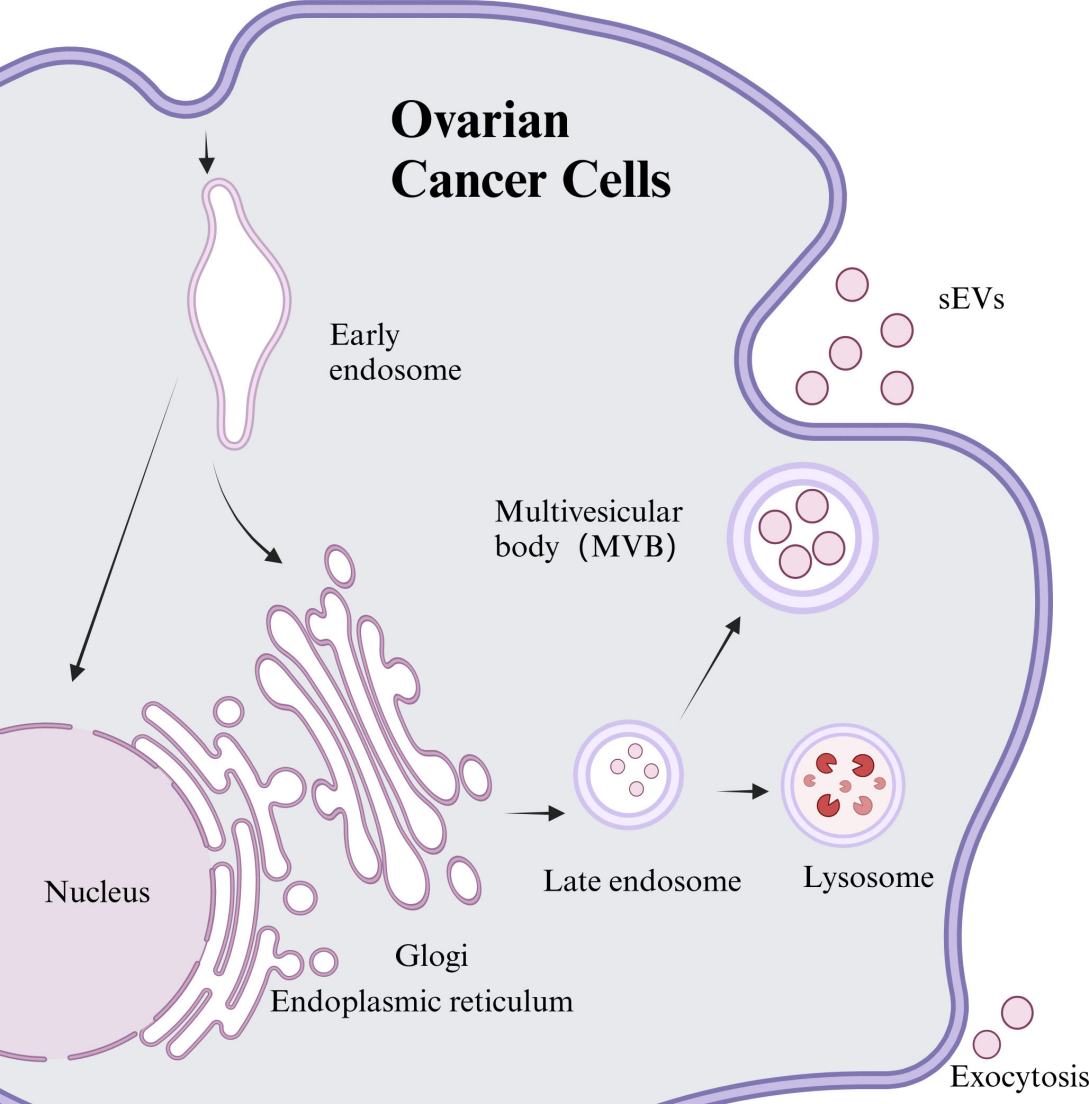
Synthesis and release of sEVs from ovarian cancer cell sources.

sEVs are lipid bilayer structures excreted by nearly all cell types and encapsulate a wide range of biologically active molecules. They were first discovered in 1983 by Johnstone et al. during *in vitro* studies of sheep reticulated erythrocytes (6). The process of sEVs generation typically occurs in three distinct phases: initially, the plasma membrane invaginates to create early endosomes; then, these early endosomes undergo further inward budding, resulting in multivesicular bodies (MVBs). Eventually, some of MVBs merge with lysosomes for degradation, while others integrate with the plasma membrane to release vesicular contents into the extracellular space, forming sEVs (7). As per the recent standards established by the International Society for Extracellular Vesicles (ISEV), this review adopts the updated terminology “sEVs” in place of the previously used proprietary term “exosomes” (8). The synthesis and secretion of sEVs can be observed in **Figure 1**.

## 3 Structural characteristics and biological functions of sEVs

Structurally, sEVs comprise a bilayer membrane structure and enclose various biomolecules. Among other functions, the bilayer membrane structure both prolongs the circulating half-life of sEVs and protects their internal proteins or nucleic acids from clearance by the complement system or macrophages, thus enhancing their biological activity (9). The content of sEVs is highly dependent on their cellular origin and includes proteins, lipids, metabolites, various RNAs (mRNAs, miRNAs, lncRNAs), and DNA fragments (10).

sEVs play a pivotal role in cell-to-cell communication, influencing a range of pathological states such as cancers and modulating the efficacy of anti-ovarian cancer drugs. Furthermore, sEVs are integral to the regulation of tumor metastasis, invasion, and proliferation. The above contents are not only involved in the development of sEVs but are also specifically recognized by receptor cells in the microenvironment, thereby facilitating the exchange of genetic material, regulation of cellular functions, and participation in cancer processes (11). For instance, enzymes like phospholipase D2 (PLD2) and certain sphingolipids, which are abundant in sEVs lipid content, are critical for releasing multivesicular bodies and stimulating sEVs secretion from tumor cells. This process enhances osteoblast activity and contributes to the establishment of bone metastasis (12). Additionally, as key components of sEVs, proteins are essential not only for their identification of sEVs and target-binding process but are also closely related to tumor development (13). Further, a variety of RNAs within sEVs hold significant potential as tumor diagnostic and prognostic markers. For example, non-coding RNAs (ncRNAs) contribute to ovarian carcinogenesis and may serve as therapeutic agents. Circular RNAs (circRNAs) offer significant value as biomarkers for diagnosis, prognosis, and treatment in ovarian cancer (14). Similarly, the long non-coding RNA (lncRNA) SPOCD1-AS, identified in ovarian cancer-derived sEVs, can induce mesenchymal transition in mesothelial cells, promoting peritoneal metastasis through interactions with the G3BP1 protein (15).

## 4 sEVs modulate the TME in EOCs

**Figure 2:**
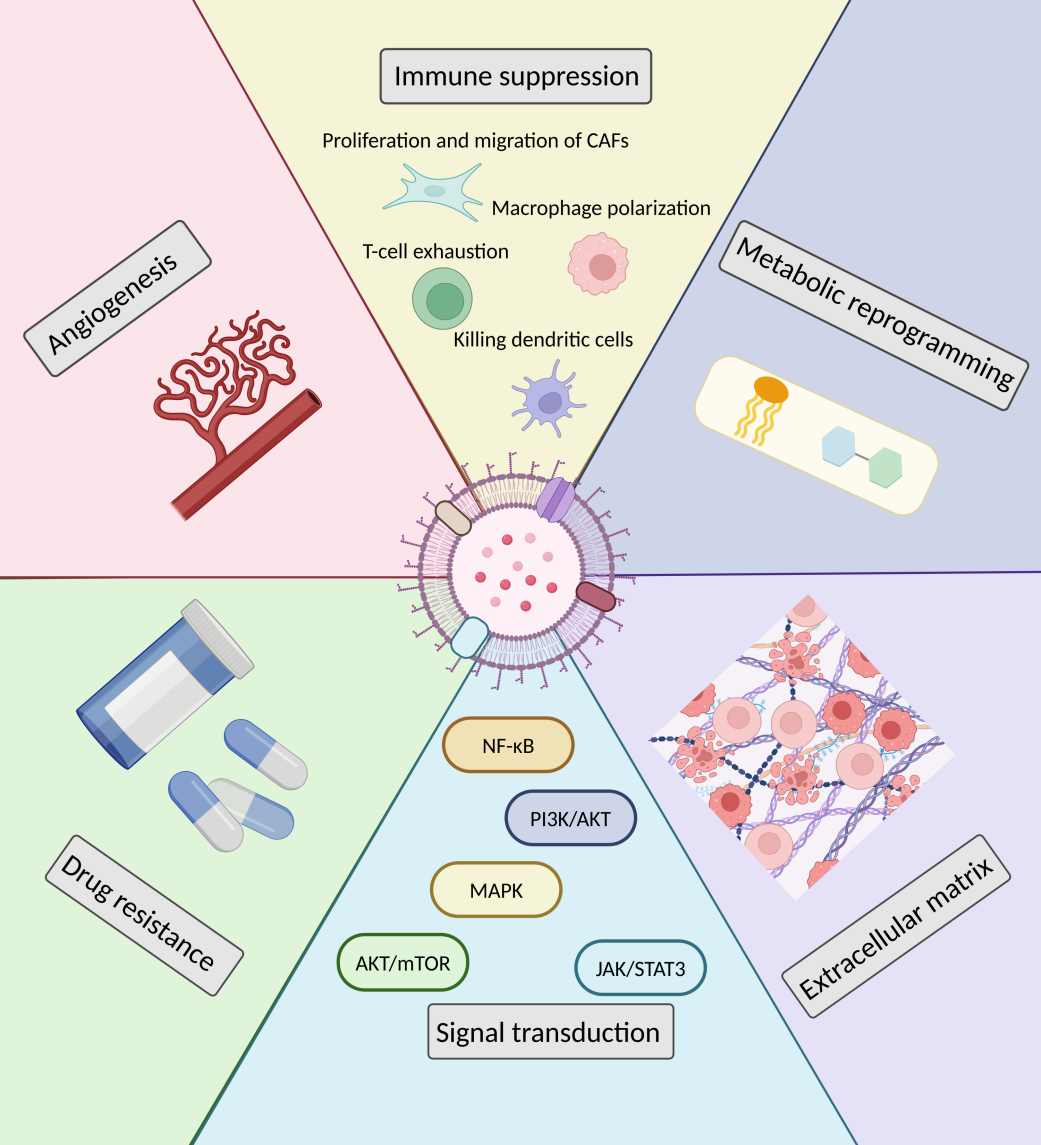
Modulation of the TME by sEVs in EOCs.

In cancer dynamics, cancer cells play a central role, while the TME also plays a crucial function, which is known as the “soil” for tumor cells to thrive (16). This internal environment of tumor cell growth is a sophisticated network comprising infiltrated immune cells, cancer-associated fibroblasts (CAFs), endothelial cells, and cytokines secreted by these cells, tissue fluids, neovasculature, and many other factors (17). Both cellular and non-cellular components of the TME interact with tumor cells, fostering tumor formation, metastasis, and leads to a poor prognosis. sEVs can shape the TME of EOCs by regulating processes such as angiogenesis, immunosuppression, signal transduction, drug resistance, and metabolic reprogramming (see **Figure 2** for details).

### 4.1 Angiogenesis

Angiogenesis, also known as neovascularization, is the biological process through which new blood vessels develop from pre-existing vascular structures. This mechanism is critical in pathological states, especially in cancer, as it supplies the necessary oxygen and nutrients required for tumor growth and spread. Li et al. demonstrated that sEVs derived from chemotherapy-resistant ovarian cancer cells notably increase the proliferation, migration, and tube formation abilities of vascular endothelial cells, with microRNA-130a (miR-130a) identified as a key regulator (18). Similarly, Liu and colleagues found that sEVs enriched with miR-205 from ovarian cancer cells affect neighboring endothelial cells by activating the PTEN-Akt signaling pathway, thus fostering angiogenesis and potentially facilitating tumor metastasis (19). Additionally, further research identified that circular RNA CircNFIX within sEVs originating from ovarian cancer cells, through the miR-518a-3p/TRIM44 axis, impacts the JAK/STAT1 signaling pathway, thereby boosting cellular proliferation, migration, and angiogenic activities. This discovery opens new avenues for targeted strategies to impede ovarian cancer progression (3).

### 4.2 Immunosuppression

In general, sEVs function as a means in mediating interactions between ovarian cancer cells and immune cells in the TME ]and promote tumor growth, advancement, and spread by modulating the functions of various immune cells to achieve immunosuppressive effects. Firstly, sEVs from ovarian cancer sources are known to influence macrophage polarization and activate the immunosuppressive macrophage M2 phenotype (20). For example, hypoxia-conditioned tumor sEVs carrying miR-1225-5p affect macrophage polarization through Toll-like receptor 2, thereby enhancing ovarian cancer’s progression (21). Secondly, sEVs secreted from tumor cells are also capable of mediating the regulation of T cell function. For instance, sphingosine kinase-1 (SPHK1) in ovarian cancer-derived sEVs promotes sphingosine-1-phosphate (S1P) synthesis in the TME and upregulates programmed death ligand 1 expression, driving T cell depletion within the TME (22). Similarly, tumor cell-derived sEVs influence CAFs proliferation and migration through the H19/miR-29c-3p/LOXL2-COL1A1 pathway (23). Third, sEVs secreted by immune cells within the TME can similarly influence other immune cells to promote tumor development, as seen with tumor-associated macrophages (TAMs). sEVs containing miR-29a-3p and miR-21-5p from TAMs together lead to a Treg/Th17 cell imbalance, aiding in ovarian cancer’s progression and metastasis (24). Moreover, miR-320a carried by sEVs from tumor cells targets the integrin subunit α7 in omental adipose-derived mesenchymal stem cells (ADSCs), shifting their phenotype towards CAFs and supporting the establishment of a pre-metastatic niche in ovarian cancer omental metastasis (25). Notably, Cocozza et al. found that extracellular vesicles (EVs) produced by a mouse tumor cell line showed distinct impacts on dendritic cell activity *in vitro*, which were sometimes opposing. Small EVs selectively trigger the death of dendritic cells. It is hypothesized that they may contain certain proteins or RNA molecules associated with immunosuppression, which may interact with receptors on the surface of dendritic cells and trigger apoptosis or necrosis. Formulations consisting of large and dense mixed EVs have proven to be the most effective at promoting the maturation of dendritic cells and enhancing their antigen-presenting capabilities (26).

### 4.3 Extracellular matrix remodeling

The sEVs encompasses a diverse array of proteins, signaling molecules, and microRNAs that play crucial roles in cytoskeletal changes, enhancing cell motility and invasiveness. Specifically, sEVs sourced from cancer cells are enriched with enzymes capable of degrading the extracellular matrix (ECM), facilitating tumor progression. These enzymes include matrix metalloproteinases (MMPs), such as MMP-2 and MMP-9, along with urokinase plasminogen activator (uPA), which aiding in ECM disintegration (27). Moreover, in patients with ovarian cancer, there is a marked increase in the expression of CD151 and Tspan8 within plasma-derived sEVs compared to those in healthy individuals. These molecules contribute to ECM breakdown and are implicated in critical functions including matrix remodeling, angiogenesis, and cell migration (28).

### 4.4 Signal transduction

Abnormalities in intracellular signaling pathways can lead to tumor development. Common signaling pathways associated with ovarian cancer include the MAPK, NF-κB, PI3K/Akt, and Akt/mTOR pathways. The contents of sEVs can act as stimulatory signals and be taken up by recipient cells, causing changes in their signaling pathways and thus promoting ovarian cancer. On one hand, ovarian cancer cells, serving as recipient cells, are capable of receiving stimulation from sEVs originating from other cell sources, prompting changes in their signaling pathways. Research has shown that secreted leukocyte protease inhibitor (SLPI) proteins, derived from CAFs, can be encapsulated in sEVs to facilitate their delivery to ovarian cancer cells. This delivery supports ovarian cancer cell proliferation, migration, invasion, and adhesion by triggering pathways such as PI3K-Akt-GSK-3β, NF-κB, and MAPK (29). Another study reported that CAFs secrete sEVs carrying miR-296-3p, which exacerbates ovarian cancer progression by targeting PTEN and SOCS6, and stimulating the Akt/STAT3 pathway (30). Furthermore, sEVs from human umbilical cord mesenchymal stem cells (MSCs) can regulate the expression of nucleus accumbens-associated protein 1 (NACC1) by transporting miR-18a-5p, which influences the Akt/mTOR pathway, thereby curbing ovarian cancer cell proliferation (31). On the other hand, ovarian cancer cells can also serve as donor cells secreting sEVs that provide stimulatory signals, whose contents are taken up by cells in the TME, causing changes in their signalling pathways. For example, epithelial ovarian cancer cells release sEVs containing miR-141-3p, which, when absorbed by vascular endothelial cells, activate the JAK/STAT3 and NFκB signalling pathways, thereby enhancing angiogenesis (32).

### 4.5 Drug resistance

Although the initial clinical response to chemotherapy in patients with intermediate to advanced ovarian cancer is usually excellent, the development of chemotherapy resistance remains a major contributor to mortality in these cases (33). Recent studies increasingly highlight that the role of sEVs is closely associated with drug resistance in ovarian cancer. Mechanistically, sEVs derived from drug-resistant ovarian cancer cell lines can confer resistance to chemotherapeutic agents on non-resistant cancer cells. For example, ADAM17, a protease known to promote tumor resistance by cleaving EGFR ligands such as AREG, is released from ovarian cancer cells via EVs following cisplatin treatment. Notably, when ADAM17-deficient cells are exposed to ADAM17-containing sEVs derived from ascites, these vesicles promote chemoresistance in ADAM17-deficient cells by triggering survival signaling pathways (34). Another example involves miR-21-5p from the sEVs of the platinum-resistant SKOV3 cell line, which, after being absorbed by the platinum-sensitive SKOV3 cells, targets PDHA1. This interaction promotes glycolysis and diminishes the chemosensitivity of the recipient cells (35). Beyond their direct impact on cancer cells, sEVs also contribute to drug resistance by interfering with immune responses. Asare-Werehene et al. found that increased secretion of sEVs containing plasma gelsolin (pGSN) from ovarian cancer cells can lead to apoptosis of CD8+ T cells and a decrease in IFNγ secretion in the TME, which in turn contributes to resistance to cisplatin (36). In addition, cells in the TME can also secrete sEVs to act on of ovarian cancer cells and subsequently support tumor drug resistance. Studies have reported that sEVs carrying GATA3 from TAMs promote tumor growth and chemoresistance via the CD24/Siglec-10 pathway (37). Moreover, sEVs rich in pGSN can alter the inflammatory microenvironment by reducing the viability and functionality of M1 macrophages, thereby contributing to chemoresistance and worsening patient outcomes (38). Interestingly, miR-296-3p encapsulated by sEVs from CAFs has been shown to regulate intercellular communication and promote ovarian cancer development by affecting the PTEN/Akt and SOCS6/STAT3 signaling pathways. High levels of miR-296-3p in sEVs are closely linked to increased chemotherapy resistance (30).

### 4.6 Metabolic reprogramming

Cells adapt their energy metabolism in response to environmental changes to adapt to the state of stress and to meet the needs of rapid cell proliferation; this adjustment is termed metabolic reprogramming (39). Research has demonstrated that sEVs released by tumor cells facilitate metabolic reprogramming. Specifically, under hypoxic conditions, ovarian cancer cells secrete an increased number of sEVs enriched with glycolysis-related proteins and other bioactive molecules. These sEVs can be taken up by cancer cells in normoxic environments, altering their metabolic pathways (such as glycolysis and fatty acid synthesis), which ultimately promotes cell survival (40). Interestingly, sEVs from various types of cells in the TME also influence the metabolism of cancer cells (41).

Furthermore, the influence of metabolic reprogramming extends beyond individual pathways, being affected by broader factors such as hypoxia and the TME (42). Under hypoxic conditions, tumor cells release sEVs to communicate with the microenvironment, thereby facilitating tumorigenesis, progression, invasion, and metastasis (43). Hypoxia has been shown to increase the secretion of sEVs containing miRNAs like miR-21-3p, miR-125b-5p, and miR-181d-5p in ovarian cancer cells. These sEVs are internalized by macrophages, where their miRNAs suppress SOCS4/5 expression and enhance STAT3 phosphorylation, inducing TAMs to polarize towards the M2 subtype and promoting tumor proliferation and migration (44). Additionally, under hypoxic conditions, miR-1225-5p within ovarian cancer-derived sEVs regulates macrophage M2 polarization via Toll-like receptor 2 (21). Moreover, hypoxic conditions enhance the secretion of sEVs exerting pro-cancer effects, while reducing the secretion of sEVs exerting tumor-suppressive effects. Similarly, the level of signal transducer and activator of transcription 3 (STAT3) in sEVs secreted by ovarian cancer cells is elevated, which may contribute to tumor progression and drug resistance. The overexpression of STAT3 can further enhance the release of pertinent sEVs by up-regulating Rab27a and down-regulating Rab27 expression, thereby creating a positive feedback cycle (45).

## 5 sEVs promote tumor metastasis in EOCs

In the TME, sEVs play a pivotal role for transmitting information. Their distinctive structure guarantees the precise delivery of signaling molecules, enhancing both the breadth and accuracy of intercellular communication. Given the profound influence of tumor heterogeneity on progression and response to treatment, sEVs are crucial in in fostering tumor heterogeneity and metastasis. Consequently, these vesicles are intimately linked with the metastatic processes of malignant tumors.

### 5.1 sEVs drive epithelial mesenchymal transition (EMT)

In ovarian cancer, peritoneal dissemination is a prevalent mode of metastasis that generally precedes vascular or lymphatic spread. EMT is a critical mechanism by which cancer cells detach from their primary location and establish new tumors in distant tissues, necessitating a phenotypic transformation that increases their motility and invasiveness. For example, sEVs from CAFs are capable of inducing EMT in ovarian cancer cell lines during co-culture, thereby enhancing their migration and invasion abilities through the activation of critical signaling pathways such as SMAD and Wnt/β-catenin (46). Additionally, sEVs present in the ascites of ovarian cancer patients are known to transport specific microRNAs, like miR-6780b-5p, which promote EMT and consequently augment the migration, invasion, and proliferation of the cancer cells (47).

### 5.2 sEVs empower tumor metastasis

Research has shown that highly metastatic ovarian cancer cells are able to transfer their migratory capabilities to less migratory cells through the secretion of sEVs. This process exemplifies how interactions among heterogeneous tumor subgroups can enhance metastasis (48). Additionally, studies on sEVs from ovarian cancer cells have uncovered a poorly characterized lncRNA that may promote peritoneal metastasis by modulating mesothelial cell phenotypes and facilitating adhesion between cancer and mesothelial cells (15). Moreover, sEVs from CAFs may convey vital signals via specific molecular pathways, such as the circIFNGR2/miR-378/ST5 axis, to regulate the metastatic behavior of ovarian cancer (49). Conversely, miR-141 in ovarian cancer-derived sEVs may mediate tumor-stroma interactions through the activation of the YAP1/GROα/CXCRs signaling cascade. This interaction promotes the establishment of a supportive TME, thereby enhancing tumor aggressiveness and increasing metastatic potential (50).

## 6 Applications of sEVs in EOCs

### 6.1 Tools for diagnosis and prognosis

The lack of effective early diagnostic markers is a major factor contributing to the less than 30% five-year survival rate for ovarian cancer, which primarily originates the deep pelvic region. Consequently, there is a critical need to identify more reliable diagnostic and prognostic markers to substantially enhance prognosis and treatment strategies. The “liquid biopsy” technique, being less invasive and capable of real-time tumor status monitoring, is anticipated to facilitate early detection of ovarian cancer. This technique encompasses circulating tumor cells (CTCs), circulating tumor DNA (ctDNA), circulating cell-free microRNAs (cfmiRNAs), and sEVs, which collectively may serve to identify ovarian cancer more effectively (51). sEVs, an integral component of the “liquid biopsy” approach, are receiving increasing attention from researchers due to their significant advantages in the diagnosis and prognosis of ovarian cancer. **Table 1** summarizes different sources of sEVs as diagnostic and prognostic markers for ovarian cancer.

**Table 1:** sEVs as diagnostic and prognostic markers for ovarian cancer.

Source	sEVs	Function	References
serum	miR-1307, miR-375	The combination of serums sEVs miR-1307 and miR-375 with CA125 improves diagnostic efficiency (AUC = 0.977).	(52)
serum	miR-34a	The expression level is linked to lymph node metastasis and recurrence.	(53)
plasma	CD24, EpCAM, FRα	Used to distinguish between ovarian cancer patients and non-cancer controls.	(54)
serum	CA125	When used in combination with plasma HE4, it enhances diagnostic efficiency.	(55)
plasma	miR-200b	The levels of miR-200b are correlated with CA125 and overall survival.	(56)
plasma	CA-125, EpCAM, CD24	The combined diagnostic efficacy of the three for ovarian cancer shows perfect performance (AUC = 1.0, *P* = 0.001).	(57)
plasma	CAV1	Low levels of CAV1 indicate a poor prognosis in ovarian cancer.	(58)
plasma, peripheral blood	wb-mtDNA copy number	Whole blood mtDNA copy numbers vary markedly between healthy individuals and those diagnosed with early or late-stage ovarian cancer.	(59)
ascites	CD24, EpCAM	sEVs released from ovarian cancer cells, which carry markers such as CD24 and EpCAM, hold potential for use in diagnostic applications.	(60)
serum	miR-484	Utilizing both serum sEVs miR-484 and CA-125 for diagnosis achieves high accuracy. Additionally, low levels of serum sEVs miR-484 are associated with a worse prognosis.	(61)
serum	miR-200a/b/c	They are effective in differentiating between benign and malignant ovarian tumors.	(62)
serum	aHIF	Increased levels of aHIF are associated with a negative prognosis in ovarian cancer.	(63)

Firstly, sEVs released into body fluids can be utilized to differentiate among cancer patients, individuals with benign ovarian tumors, and healthy individuals. Jo et al. identified high-grade serous ovarian cancer (HGSOC)-specific extracellular vesicle markers by establishing fallopian tube tumor cells and performing proteomic analysis. The combined expression of these markers—EpCAM, CD24, VCAN, HE4, and TNC—has demonstrated the ability to distinguish between non-cancerous, early, and advanced HGSOC, offering the possibility of a non-invasive monitoring program for women at high risk (64). Serum sEVs containing miR-1307 and miR-375 were found to be significantly elevated in ovarian tumors compared to benign tumors and healthy ovarian groups. When combined with the cancer marker CA125, the levels of miR-1307 and miR-375 in serum sEVs improved diagnostic accuracy, achieving an Area Under the Curve (AUC) of 0.977. Additionally, miR-1307 was associated with tumor stage, while miR-375 was correlated with lymph node metastasis in ovarian cancer (52).

Secondly, significant distinctions between early and advanced ovarian cancer were noted in plasma sEVs, aiding in disease assessment. For example, levels of miR-34a in serum-derived sEVs were markedly higher in early-stage patients than in those with advanced ovarian cancer, and significantly lower in patients with lymph node metastasis and in the recurrence group compared to their respective controls (53). A study identified six new proteins in sEVs — ACSL4, IGSF8, ITGA2, ITGA5, ITGB3, and MYOF — that, in combination with a previously recognized protein associated with HGSOC, can distinguish between plasma samples from early-stage (including stage IA/B) and advanced-stage (stage III) HGSOC. Crucially, these proteins also demonstrated the capability to accurately differentiate between ovarian cancer and twelve other cancers commonly diagnosed in women. This suggests the potential for these markers to be used in a non-invasive, highly specific blood test that could facilitate pre-symptomatic screening and early detection of ovarian cancer (65). The diagnostic utility of sEVs-based biosensors, specifically the sEVs/CA125 ratio, has shown superior diagnostic value compared to CA125 or sEVs alone in detecting stage I disease, significantly enhances the diagnostic efficiency for ovarian cancer (66).

Finally, sEVs in body fluids have demonstrated prognostic value, enabling the prediction of patient outcomes. A risk prediction model incorporating a 4-miRNA signature and CA-125 from plasma sEVs showed excellent calibration and discriminative ability in predicting R0 resection, achieving an AUC of 0.903, and this combined model significantly outperformed CA-125 or the 4-miRNA signature alone (67). Notably, Asare-Werehene et al. highlighted that the sEVs/CA125 ratio excels in forecasting tumor recurrence, chemotherapy resistance, residual lesions, and patient survival, making it a promising pre-treatment or pre-surgery prognostic tool (66). Furthermore, miR-141-3p and miR-200c-3p, extracted from serum-derived sEVs, have been pinpointed as promising prognostic indicators, displaying notable variations across different groups of ovarian cancer patients, including those with preoperative benign conditions, preoperative malignant conditions, postoperative complete resection (R0), and postoperative residual disease. The sEV miR-EOC model appears to offer substantial promise for forecasting the prognosis of ovarian cancer (68).

However, a number of challenges are associated with the current utilization of sEVs as a diagnostic tool. For instance, research on sEVs diagnostics remains at the experimental stage, lacks large-scale clinical validation, and detection is challenging due to their size and phenotypic heterogeneity (69). Therefore, how to efficiently extract and identify sEVs in body fluids and apply them to the diagnosis of ovarian cancer remains a key focus of current research. Zhang P et al. designed a 3D-nano-forming microfluidic chip (Nano-HB Chips) capable of ultrasensitive detection of low levels of tumor-associated sEVs in circulating blood. The device was employed to identify sEVs in the blood samples from 20 ovarian cancer patients and 10 non-cancerous controls. In both groups, sEVs were successfully detected using this device. Importantly, the presence of folate receptor α in sEVs emerged as a potential marker for the early detection and monitoring of ovarian cancer progression (54). Vaidyanathan et al. introduced a chip-based extended nanocoulter counter (XnCC) capable of detecting affinity-selected nanoparticles from biological samples at low concentration thresholds. Using the XnCC technique, ovarian cancer derived sEVs were selectively captured on a column chip coated with a MUC16 monoclonal antibody, enabling differentiation between HGSOC patients and healthy individuals (70). In a separate study, Li et al. implemented an aptamer-based nano-flow cytometry (nFCM) approach. This method swiftly analyzed seven cancer-associated protein markers—CA125, STIP1, CD24, EpCAM, EGFR, MUC1, and HER2—on plasma sEVs. The nFCM achieved precise detection of ovarian cancer and accurately classified five ovarian cancer subtypes with a 94.2% accuracy rate (71).

### 6.2 Innovations in treatment

Adjuvant chemotherapy following primary tumor cytoreduction is established as the standard treatment for ovarian cancer (72). The pursuit of precision in ovarian cancer drug therapy, specifically targeted drug delivery and controlled release, represents a core focus within the ovarian cancer drug research domain. While liposomes and polymer nanoparticles can enhance drug delivery specificity, extend drug release, and mitigate drug degradation, these synthetic carriers also present significant drawbacks, including potential organ toxicity and immune reactions (73). In contrast, sEVs feature a biocompatible lipid bilayer that prevents their clearance and degradation in the circulation, enabling them to effectively reach the target area. Moreover, sEVs exhibit exceptional biocompatibility, minimal toxicity, and immunogenicity as carriers, alongside their capability for targeted transportation (74).

Research has utilized sEVs as a method for precise delivery of chemotherapy drugs in treating ovarian cancer. Utilizing sEVs as carriers can greatly reduce the adverse effects and toxicity typically linked to drugs. Particularly, sEVs extracted from expanded natural killer cells (eNK-EXO) demonstrate a capacity to eliminate SKOV3, OV90, and COC1/DDP ovarian cancer cells based on dosage, while sparing normal cells from cytotoxic impacts. These sEVs can also serve as delivery vehicles for the chemotherapeutic agent cisplatin, augmenting its cytotoxic impact on drug-resistant ovarian cancer cells and overcoming the immunosuppression of NK cells, thus offering considerable potential for ovarian cancer treatment in clinical settings (75). Li et al. developed a novel sEVs-based hydrogel targeted at peritoneal macrophages, presenting a new therapeutic approach for managing ovarian cancer. This hydrogel utilizes artificially engineered sEVs derived from genetically modified M1-type macrophages, featuring salivary acid-conjugated Ig-like lectin 10 (Siglec-10), as the gelling agent. This approach also shows efficacy in treating intrinsic CD24 overexpressing triple-negative breast cancer (76). The triptolide-loaded sEVs delivery system (TP-Exos) has been shown to induce apoptosis in ovarian cancer cells, regulating tumor immunity through the activation of the mitochondrial apoptotic pathway, and selectively targeting M2 TAMs along with their tumor-promoting factors in the TME (77).

Furthermore, sEVs capable of carrying drugs into the TME have been proven to improve the effectiveness of therapeutic approaches against ovarian cancer. Studies indicate that sEVs sourced from M1-type macrophages in umbilical cord blood and loaded with cisplatin substantially enhance the cytotoxic effects on drug-resistant ovarian cancer strains (78). Drug resistance is a major obstacle in treating advanced ovarian cancer, yet sEVs have shown effectiveness in overcoming tumor drug resistance. Tumor-derived sEVs are capable of effectively transporting CRISPR/Cas9 plasmids to tumor tissues, suppressing the expression of poly (ADP-ribose) polymerase-1 (PARP-1). This inhibition triggers apoptosis and enhances the sensitivity of ovarian cancer cells to the chemotherapeutic agent cisplatin (79). Noteworthy is the targeted delivery of alternative molecules via sEVs for ovarian cancer treatment, which has emerged as a research focal point in recent years. For instance, microRNAs functioning as cancer inhibitors have demonstrated the capability to exert anti-cancer effects *in vivo* through sEVs. sEVs from fibroblasts loaded with miR-199a-3p (miR-199a-3p-Exo), harvested from primary cultured omental tissues of ovarian cancer patients, have been shown to inhibit peritoneal dissemination of tumors by repressing the expression of the target gene c-Met (80).

Nonetheless, significant challenges persist in the realm of sEVs-targeted drug delivery research. The foremost challenge for researchers is the production, isolation, and purification of sEVs capable of transporting drugs in substantial quantities. Recent progress in bioengineering and nanotechnology has facilitated the incorporation of various therapeutic agents into tumor-targeted sEVs, with the practicality of this approach being exemplified through the use of artificial sEVs targeting peritoneal macrophages for ovarian cancer therapy (76). Furthermore, the delivery of natural compounds exhibiting notable anticancer properties, such as curcumin and tretinoin lactoneol, is also under explored (81). An ongoing clinical trial is presently investigating the delivery of curcumin through plant-derived sEVs to both colon tumors and normal colon tissues. There is an imperative need to formulate standardization of both qualitative and quantitative methodologies to enhance the successful commercialization of sEVs within the field of translational medicine (82).

## 7 Summary

The TME is crucial in tumorigenesis, and sEVs, as signaling molecules for cell-cell interactions, are specifically recognized and utilized by receptor cells within this environment. sEVs can shape the TME in epithelial ovarian carcinogenesis through energy regulation, provision of stimulatory signals, immunosuppression, and other mechanisms. These functions underscore their critical influence in both altering the TME and advancing epithelial ovarian carcinogenesis. Current discoveries suggest that sEVs has been proved to be an effective biomarker and therapeutic target for EOCs. The further development of new drugs related to sEVs or the search for new diagnostic markers might offer promising prospects for pioneering early diagnostic techniques and therapeutic strategies for EOCs.
